# Zika virus: An updated review of competent or naturally infected mosquitoes

**DOI:** 10.1371/journal.pntd.0005933

**Published:** 2017-11-16

**Authors:** Yanouk Epelboin, Stanislas Talaga, Loïc Epelboin, Isabelle Dusfour

**Affiliations:** 1 Vectopôle Amazonien Emile Abonnenc, Vector Control and Adaptation Unit, Institut Pasteur de la Guyane, Cayenne, French Guiana, France; 2 Infectious and Tropical Diseases Unit, Centre Hospitalier Andrée Rosemon, Cayenne, French Guiana, France; 3 Ecosystèmes amazoniens et pathologie tropicale (EPAT), EA 3593, Université de Guyane–Cayenne, French Guiana; Colorado State University, UNITED STATES

## Abstract

Zika virus (ZIKV) is an arthropod-borne virus (arbovirus) that recently caused outbreaks in the Americas. Over the past 60 years, this virus has been observed circulating among African, Asian, and Pacific Island populations, but little attention has been paid by the scientific community until the discovery that large-scale urban ZIKV outbreaks were associated with neurological complications such as microcephaly and several other neurological malformations in fetuses and newborns. This paper is a systematic review intended to list all mosquito species studied for ZIKV infection or for their vector competence. We discuss whether studies on ZIKV vectors have brought enough evidence to formally exclude other mosquitoes than *Aedes* species (and particularly *Aedes aegypti*) to be ZIKV vectors. From 1952 to August 15, 2017, ZIKV has been studied in 53 mosquito species, including 6 *Anopheles*, 26 *Aedes*, 11 *Culex*, 2 *Lutzia*, 3 *Coquillettidia*, 2 *Mansonia*, 2 *Eretmapodites*, and 1 *Uranotaenia*. Among those, ZIKV was isolated from 16 different *Aedes* species. The only species other than *Aedes* genus for which ZIKV was isolated were *Anopheles coustani*, *Anopheles gambiae*, *Culex perfuscus*, and *Mansonia uniformis*. Vector competence assays were performed on 22 different mosquito species, including 13 *Aedes*, 7 *Culex*, and 2 *Anopheles* species with, as a result, the discovery that *A*. *aegypti* and *Aedes albopictus* were competent for ZIKV, as well as some other *Aedes* species, and that there was a controversy surrounding *Culex quinquefasciatus* competence. Although *Culex*, *Anopheles*, and most of *Aedes* species were generally observed to be refractory to ZIKV infection, other potential vectors transmitting ZIKV should be explored.

## Author summary

The first isolation of Zika virus (ZIKV) in mosquitoes was made in 1948 in *Aedes africanus*. Over the next years, knowledge about ZIKV increased, with detection of the virus in primates, including humans and several other mosquito species. Most of these species were collected in Africa during arbovirus surveillance studies and belong to the genus *Aedes*, and today, 20 mosquito species have been identified that can be naturally infected by ZIKV. Although field studies are essential to have an overview of potential mosquito vectors of ZIKV during outbreaks or involved in the maintenance of the sylvatic cycle, laboratory studies are needed to assess the capacity of a species to transmit the virus to a new host. Since 2015, corresponding to the beginning of the outbreak in Brazil, vector competence studies have multiplied and confirmed that the mosquito *A*. *aegypti*, known to transmit dengue fever and chikungunya viruses, was also the main vector of ZIKV. This review aims to highlight the studies conducted from several laboratories about mosquito species naturally infected or tested for their vector competence for ZIKV.

## Introduction

Zika virus (ZIKV) is an arthropod-borne virus (arbovirus) belonging to the family Flaviviridae and the genus *Flavivirus*. It is a single-stranded RNA virus that was first isolated in 1947 from a sentinel rhesus monkey in the Zika Forest in Uganda and in 1948 from *A*. *africanus* mosquitoes in the same forest, suggesting the mosquito-borne transmission of the virus [1]. Over the past 60 years, this virus has been observed circulating among African and Asian populations [2] but with little attention from the scientific community. The ZIKV lineage circulating in Asia has been described as distinct from the African lineage, suggesting the separate sylvatic cycles of ZIKV on those continents [3]. In 2007, the first ZIKV outbreak occurred on Yap Island of the Federal States of Micronesia [4]. Between 2013 and 2014, French Polynesia was struck by ZIKV, with, for the first time, Guillain-Barré syndrome reported in a few patients following ZIKV infection. ZIKV then spread to several islands of the Pacific Ocean [5,6]. This virus may have been subsequently introduced into Brazil, but the origin of this introduction remains uncertain, and several hypotheses have been proposed, all related to international travel. These hypotheses include the visit of the Pope, with many young Catholics from Africa and Asia visiting Brazil during World Youth Day in July 2013, the World Cup in 2014 gathering thousands of people in stadiums and in various regions of Brazil, and the canoeing championship in Rio de Janeiro in 2014, with participants from Pacific countries in which ZIKV circulated during 2014 [7]. Currently, the virus circulating across the Pacific and South America belongs to the Asian lineage [3]. There has been an increasing interest in ZIKV since the outbreak started in Brazil in 2015 and spread into most of the countries of South and Central America, as well as Florida in the United States, with evidence that infection by ZIKV is associated with neurological complications such as microcephaly and several other neurological malformations in fetuses and newborns [8–10]. Current evidence is that ZIKV is not only transmitted by the bite of an infected mosquito but also through sexual intercourse [11–13]. However, mosquito bites remain the predominant route of virus transmission, with an incubation period of about 9 days and then the onset of symptoms [11].

Presently, various facts incriminate the mosquito *A*. *aegypti* as the main vector of ZIKV, given that it has been shown to be competent in transmitting this virus. This species is the main vector of dengue fever virus (DENV), chikungunya virus (CHIKV), and yellow fever virus. It originated in Africa and spread to Neotropical areas in the 17th and 18th centuries. Urbanization is the main factor, which facilitates an increase in *A*. *aegypti* populations through the proliferation of human-made containers used to store water in and around inhabited areas, which provide the aquatic larval environment required by these mosquitoes. For this reason, *A*. *aegypti* appears to be predominant in the transmission and spread of the virus during the recent ZIKV outbreak. Facing the strength and spread of the South American ZIKV epidemics and the fact that *A*. *aegypti* is not the only mosquito species living in epidemic areas, other species have been suspected to transmit the virus. Moreover, a mosquito that would transmit ZIKV needs to feed on a viremic person and become infected, and the virus must disseminate to the hemocoel, infect the salivary glands, and be secreted into the saliva (this is the vector competence portion of vectorial capacity). That same mosquito must then feed on one or several other person(s) to disseminate ZIKV into the population. For most species of mosquitoes other than *A*. *aegypti* (and a few closely related species), this would be extremely unlikely because of their lower anthropophilic behavior but would need to be accounted for in determining how that species would be an important vector. This information is critical when determining whether a particular mosquito species can be a vector of epidemiological importance, and that is why studies mainly focused on *A*. *aegypti* and *C*. *quinquefasciatus* because they are the most abundant urban mosquitoes in South America and because they preferentially feed on humans. Moreover, *Culex* mosquitoes are known to transmit several viruses from the same viral family as ZIKV, such as the West Nile virus or the Japanese encephalitis virus, and current knowledge about *Culex* species needs to be examined with greater consideration. This paper is a systematic review intended to evaluate whether studies performed on ZIKV vectors have brought enough evidence to formally exclude mosquitoes other than the *Aedes* species (and particularly *A*. *aegypti*) as ZIKV vectors. To that end, we discuss the well-established and potential vector competence and capacity of various mosquito species. The relevance of looking more closely at the role played by the mosquito *Ae*. *aegypti*, which was considered the main vector of Zika during the American outbreak, was highlighted. The assumption that other mosquito species could have been involved in the past or are involved in current epidemics is reviewed, focusing on vector competence and mosquitoes naturally infected by the virus.

## Methods

This study was conducted using the electronic databases PubMed and ScienceDirect with a cutoff date of August 15, 2017. Search terms included “Zika” and one of the following search items: “vector,” “mosquito,” “Culicidae,” “Aedes,” “Culex,” and “Anopheles.” Articles reviewed for this study were exclusively in English and French, in addition to some articles obtained through classical search engines because they were not referenced in the above-mentioned databases. After screening the abstracts, titles, and keywords of the identified citations, ineligible articles that mainly focused on outbreaks and the ensuing issues for humans were discarded. Eligible articles were considered relevant if they mentioned one or more aspects of the research question (i.e., entomological studies), regardless of when or where the studies were conducted.

To incriminate a species as a pathogen vector, different criteria are merged into the term “vectorial capacity,” which describes the dynamic relationship between the vectors of infectious diseases and vertebrate hosts, including environmental parameters [14]. The primary components used in estimating the vectorial capacity of mosquitoes are vector density in relation to host density, host preference, and host feeding patterns. In addition, daily survival rates and the longevity of mosquitoes are used, as well as their extrinsic incubation period and vector competence, which is the intrinsic ability (genetic) of a species (mosquito) to be infected, multiply, and transmit a pathogen to another host [15].

Herein, the cited papers used the following parameters. The infection rate corresponds to the proportion of mosquitoes with virus-infected bodies among those tested. The dissemination rate corresponds to the proportion of mosquitoes with virus-infected legs or heads among those infected. The transmission rate corresponds to the proportion of mosquitoes with infectious saliva among those infected. Transmission efficiency corresponds to the proportion of mosquitoes with infectious saliva among those tested.

The validity of species determination was based on the “Systematic Catalog of Culicidae” provided by the Walter Reed Biosystematics Unit [16], and the abbreviations for genus follow the recommendations of Reinert [17]. Considering that there is no consensus concerning the internal classification of the Aedini tribe proposed by Reinert et al. [18], in the current revision, we decided to use the traditional classification for Aedini.

## Results and discussion

### Publication result overview

**Fig 1:**
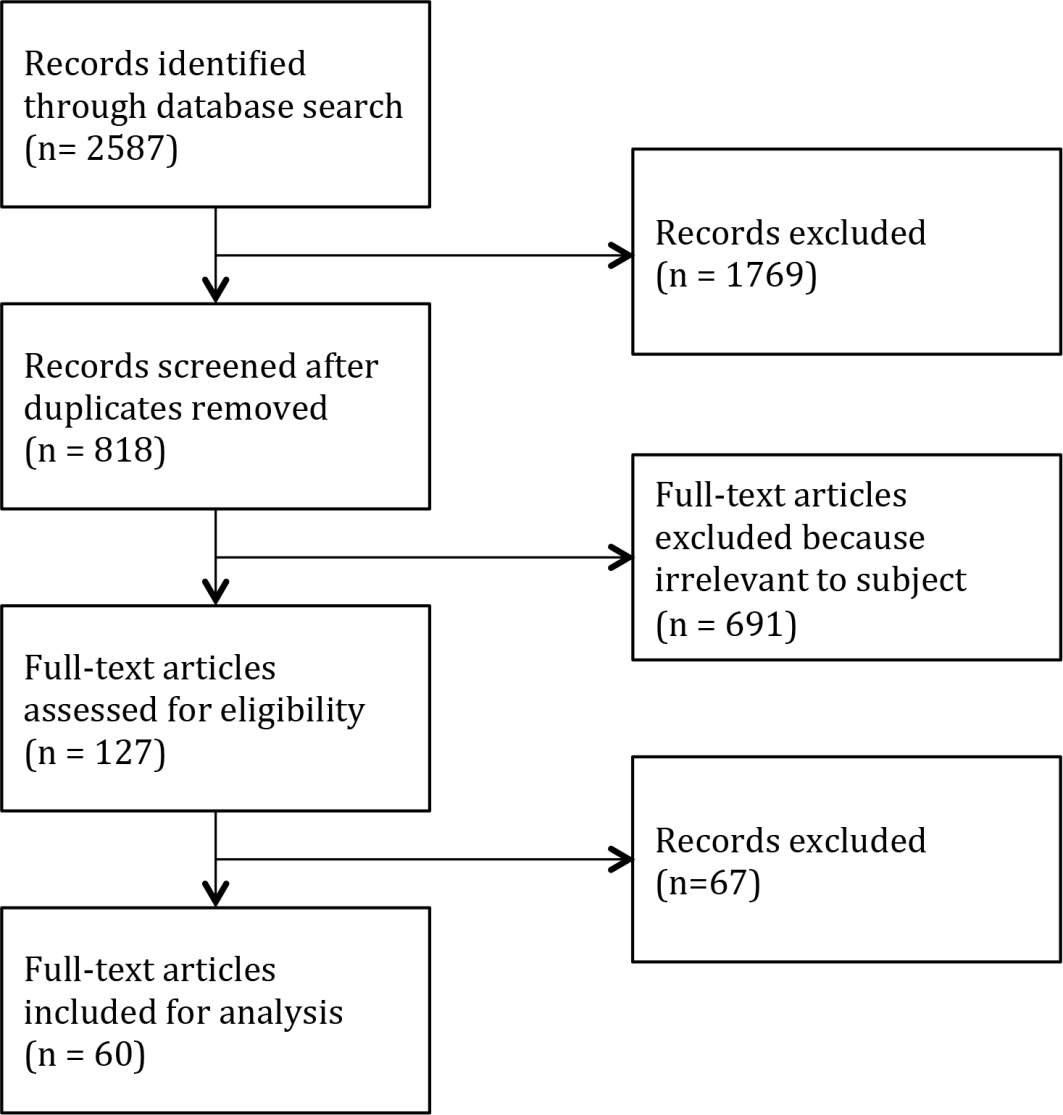
Flow diagram of search strategies for eligible studies.

**Fig 2:**
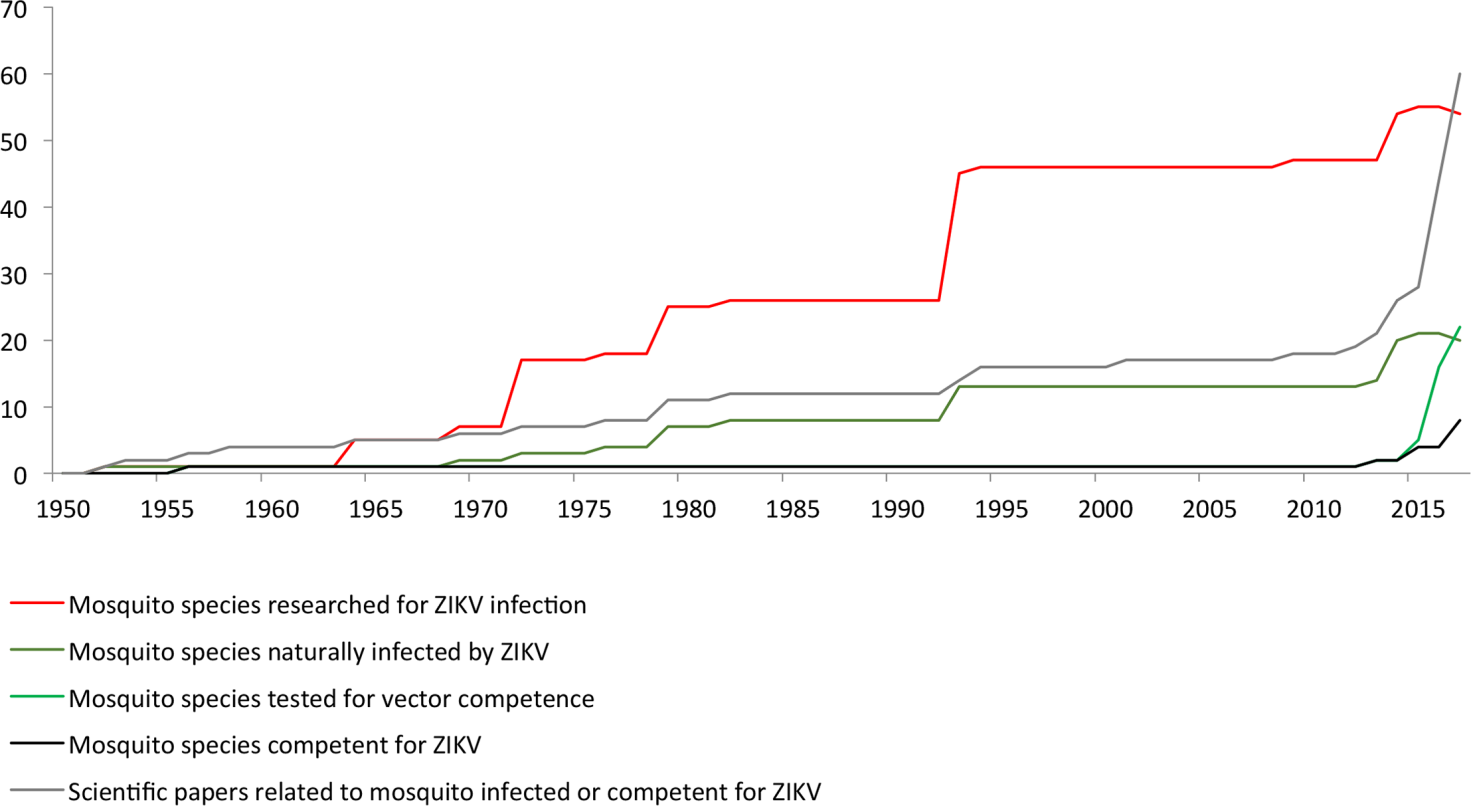
Synthesis of the research related to vector species of ZIKV between 1952 and March 15, 2017. Evolution of the number of scientific papers related to vector species of ZIKV, the number of field species tested for the presence of Zika, the number of naturally ZIKV-infected species, the number of species studied experimentally for their competence, and the number of species observed once competent for ZIKV between 1952 and March 15, 2017. ZIKV, Zika virus.

The Boolean search identified 562 articles for “Zika vector,” 865 for “Zika mosquito,” 398 for “Zika culicidae,” 654 for “Zika Aedes,” 73 for “Zika Culex,” and 35 for “Zika Anopheles.” Among these 2,587 records, after eliminating duplicates and irrelevant records, 127 studies were considered eligible, finally resulting in 60 considered relevant to this review (Fig 1). Among these 60 articles, 37 were related to ZIKV vector competence and 23 corresponded to mosquitoes naturally infected in the field (Fig 2). From 1952 to 2017, ZIKV has been searched in 1 tabanid species and 53 mosquito species, including 6 *Anopheles*, 26 *Aedes*, 11 *Culex*, 2 *Lutzia*, 3 *Coquillettidia*, 2 *Mansonia*, 2 *Eretmapodites*, and 1 *Uranotaenia* (Table 1). Among those, ZIKV was isolated from 16 different *Aedes* species. The only species other than *Aedes* for which ZIKV was isolated were *A*. *coustani*, *A*. *gambiae*, *C*. *perfuscus*, and *M*. *uniformis* [19–21]. Vector competence assays were performed on 22 different mosquito species, including 13 *Aedes*, 7 *Culex*, and 2 *Anopheles* species (Fig 2 and S1 Table).

**Table 1:** Vector species naturally infected by ZIKV or studied experimentally for their ZIKV competence.

Vector species	Vector competence assay		Field studies with natural ZIKV infection	
	Competent	Not competent	Infected	Not infected
**Culicidae: Anophelinae**				
*Anopheles brohieri*				[20]
*Anopheles coustani*			[19,21]	[20,22]
*Anopheles funestus*				[20,22,23]
*Anopheles gambiae*		[24]	[21]	[20,22,23,25]
*Anopheles nili*				[20,22]
*Anopheles rufipes*				[21,26]
*Anopheles stephensi*		[24]		
**Culicidae: Culicinae: Aedini**				
*Aedes aegypti*	[27–53]	[54,55]	[19,21,26,56–59]	[22,23,25,60,61]
*Aedes africanus*			[1,19,21,62–67]	[22,23,68]
*Aedes albopictus*	[30,32,35,42,46–48,51,69]		[25]	[56,58,59]
*Aedes apicoargenteus*			[65]	
*Aedes argenteopunctatus*				[20–22,60]
*Aedes camptorhynchus*	[51]			
*Aedes circumluteolus*				[22]
*Aedes cozi*				[60]
*Aedes cumminsii*				[22,60]
*Aedes dalzieli*			[19–21,26,60]	
*Aedes fowleri*			[21,26]	[22]
*Aedes furcifer*			[19,21,22,26,57,60,67]	
*Aedes hensilli*				[4,61]
*Aedes hirsutus*			[19]	[22]
*Aedes ingrami*				[63]
*Aedes luteocephalus*	[55]		[19–23,26,60,67]	
*Aedes metallicus*			[19,21]	[22]
*Aedes minutus*			[21,26]	[20]
*Aedes neoafricanus*			[21,26]	[20,60]
*Aedes notoscriptus*	[51]	[38]		
*Aedes opok*			[22,66,68]	[57,60]
Aedes palpale*				[22]
*Aedes polynesiensis*		[31]		
*Aedes procax*		[38]		
*Aedes simpsoni*				[23,25]
*Aedes taeniorhynchus*		[70]		
*Aedes tarsalis*				[22]
*Aedes taylori*			[19,21,26]	[60]
*Aedes triseriatus*		[35]		
*Aedes unilineatus*		[55]	[19]	[22,60]
*Aedes vexans*	[71,72]			[61]
*Aedes vigilax*		[38]		
*Aedes vittatus*	[55]		[19–21,26,57]	[22,23,60]
**Culicidae: Culicinae: Culicini**				
*Culex annulioris*				[22,63]
*Culex annulirostris*		[38,51]		
Culex bitaeniorhynchus*				[21,26]
*Culex cinereus*				[22]
*Culex duttoni*				[23]
*Culex gossi*				[61]
*Culex molestus*		[42]		
*Culex nebulosus*				[23]
*Culex nigropunctatus*				[61]
*Culex perfuscus*			[19]	[26]
*Culex pipiens*		[33,35,39,42,49,73,74]		
*Culex poicilipes*				[21,22]
Culex quinquefasciatus*	[37,75]	[24,36,38,39,43,46,48, 49,51,70,73,74]		[49], [50], [55], [61], [63], [64]
*Culex sitiens*		[38]		[61]
*Culex tarsalis*		[39]		
*Culex torrentium*		[42]		
*Lutzia fuscana*				[61]
Lutzia tigripes*				[23]
**Culicidae: Culicinae: Mansoniini**				
Coquillettidia aurites*				[63]
*Coquillettidia crassipes*				[61]
*Coquillettidia cristata*				[22]
*Mansonia africana*				[21,22,25]
*Mansonia uniformis*			[19–21]	[23,25]
**Culicidae: Culicinae: Sabethini**				
*Eretmapodites chrysogaster*				[22]
*Eretmapodites quinquevittatus*				[25]
**Culicidae: Culicinae: Uranotaeniini**				
*Uranotaenia balfouri*				[21]
**Tabanidae**				
*Chrysops centurionis*				[63]

In the literature cited, samples with pools containing more than 1 species were excluded. Species are ranked alphabetically by family, subfamily, tribe, and genus.

*Aedes palpale was formerly called *Aedes palpalis*, *Culex quinquefasciatus* was formerly called *Culex pipiens fatigans*, *Lutzia tigripes* was formerly called *Culex tigripes*, *Culex bitaeniorhynchus* was formerly called *Culex ethiopicus*, and *Coquillettidia aurites* was formerly called *Mansonia aurites*.

Abbreviation: ZIKV, Zika virus.

### Mosquitoes tested and naturally infected with ZIKV

#### In Africa

Historically, the first detections of ZIKV in mosquitoes were observed in species of the genus *Aedes* in studies carried out in Africa through methods of immunoassays for detection of antiviral antibodies (mostly hemagglutination inhibition test). The first isolation of ZIKV occurred in 1947 during a routine surveillance for yellow fever in the Zika Forest, Uganda, from a sentinel rhesus monkey [1]. The following year, in 1948, this virus was detected from *A*. *africanus*, a sylvatic mosquito, by intracerebral neutralization test in the same forest [1]. The use of defined antisera in these tests permitted differentiation of ZIKV from other known viruses such as yellow fever or DENV. Over the next 10 years, knowledge about ZIKV increased. Primates, including humans, were considered to be the main reservoirs of ZIKV, with transmission to humans primarily through mosquito vectors [1,63,76]. In the interim, several arboviruses were isolated in various mosquitoes from the Zika Forest, including *Aedes ingrami* and *Culex annulioris*, but ZIKV remained identified only in *A*. *africanus* (Table 1) [62,63]. The first isolation in a species other than *A*. *africanus* occurred in 1969 in *A*. *aegypti* from Malaysia [56]. In that study, *A*. *albopictus* was also sampled but did not exhibit ZIKV infection. However, by 2000, ZIKV was detected from 13 distinct mosquito species among the 43 species studied in several African countries (Fig 2 and Table 1). During a yellow fever outbreak in Nigeria, *Aedes luteocephalus* was shown to be infected by ZIKV. Surprisingly, in that study, neither *A*. *africanus* nor *A*. *aegypti* exhibited the presence of ZIKV [23]. However, the primary vector of ZIKV was suspected to be *A*. *aegypti*, first because of its urban habitats concomitant with the high prevalence observed in human populations [77] and also because its ability to transmit the virus to a new host was shown experimentally [28].

Several arbovirus surveillance studies conducted in Africa highlighted an increasing number of mosquitoes naturally infected with ZIKV. Thus, in the Central African Republic, ZIKV was isolated from *Aedes opok* and *A*. *africanus* [64,68]. In Senegal, *Aedes furcifer*, *Aedes taylori*, *A*. *luteocephalus*, *Aedes dalzieli*, *Aedes vittatus*, and *M*. *uniformis* were infected by ZIKV [20], as were *A*. *aegypti*, *A*. *africanus*, *Aedes neoafricanus*, *Aedes fowleri*, *Aedes metallicus*, and *Aedes minutus* [21,26,60]. In the latter study, ZIKV was also isolated from *A*. *coustani*, *A*. *gambiae*, and *M*. *uniformis* [21]. In Burkina Faso and Ivory Coast, ZIKV was isolated from various *Aedes* species [22,57]. In 2014, another study conducted in Senegal revealed that several *Aedes* species were naturally infected by ZIKV, as were *C*. *perfuscus* and *M*. *uniformis*, suggesting that ZIKV was still circulating in Africa [19]. ZIKV isolated from *Culex*, *Anopheles*, or *Mansonia* species might reveal their potential role as secondary vectors in the transmission and viral maintenance of ZIKV. However, based on recent studies revealing that neither *C*. *quinquefasciatus* nor *Culex pipiens* can be considered competent species [73,74], it seems more plausible that this infection was caused by an undigested blood meal after a bite on animals infected with ZIKV. Some nonvector species could also develop gut-limited infections without transmitting the pathogen [78]. Vector competence studies are nevertheless not consensual concerning the ability of these 2 species to be infected and to transmit ZIKV, suggesting that *Culex* species but also other potential vectors transmitting ZIKV should be explored [37,75]. A study performed in Gabon in 2007 isolated ZIKV in *A*. *albopictus*, revealing a new potential threat from this invasive species in Africa but also out of Africa [25]. Although ZIKV hosts are not clearly identified, the ZIKV transmission cycle involves one or more vertebrate hosts and one or more mosquito vectors. In Africa, ZIKV is mainly maintained by a sylvatic cycle involving nonhuman primates; however, some serological studies suggest that other mammals could be reservoirs [79].

#### Out of Africa

Between the end of the 1960s and the 2000s, ZIKV dispersed to several Asian countries, as observed through seroprevalence studies [80], but few vector studies were conducted, possibly because of the clinical similarity of the symptoms of ZIKV, DENV, and CHIKV infections. In 2007, after the ZIKV outbreak reported in the Pacific Island of Yap, entomological studies mainly focused on *Aedes hensilli* because of its abundance and the presumption that it was the most likely vector of DENV. Even if 73% of Yap residents were estimated to have been recently infected by ZIKV, the virus was not isolated from the sampled *A*. *hensilli* or *C*. *quinquefasciatus* [4,61]. However, high rates of infection were found in a study in which the probable vector *A*. *hensilli* was experimentally infected, reinforcing the plausibility that this species served as a vector during the ZIKV outbreak [61]. *A*. *aegypti* nevertheless remained the suspected vector in the transmission of ZIKV in Asia.

In 2013–2014, ZIKV caused outbreaks in several Pacific Islands including French Polynesia and Easter Island in Chile, but no vector was strictly identified, although *A*. *aegypti* and *Aedes polynesiensis* were assumed to play a role in the transmission of the virus [81,82].

In 2015, some cases of humans infected by ZIKV were reported in Brazil, developing into an outbreak that spread throughout South America, the Caribbean islands, and Central America. Originally adapted to a zoonotic cycle in Africa, ZIKV evolved into an urban cycle involving a human reservoir and domestic mosquito vectors. In South America, *A*. *aegypti* and *C*. *quinquefasciatus* are among the most abundant species in urban areas and were first suspected to be the main vectors of ZIKV. *C*. *quinquefasciatus* is both a domestic and opportunistic mosquito in its feeding behavior. Because it does not have a marked trophic preference, the blood meals it takes are largely conditioned by the host populations [83]. In South American urban areas, the community of vertebrates is dominated by humans. In this case, the probability that one of these *Culex* takes 2 consecutive blood meals on a human more than 7 days apart is high and could imply a significant role of this species in the transmission of ZIKV. Consequently, at the beginning of the outbreak in the Americas, the questions surrounding the detection of ZIKV only in *Aedes* species and not in other species remain: was this due to a lack of data, or did this reflect the reality of the situation?

### Vector competence evidence

#### A. aegypti

The first evidence of ZIKV transmission via mosquito bites was found experimentally for an African strain of *A*. *aegypti* in 1956 in Nigeria; even though *A*. *africanus* was presumed to be the vector, the number of samples was insufficient at that time [27]. Although *A*. *aegypti* was not primarily suspected to be involved in the transmission of ZIKV, that study demonstrated that the virus was probably able to multiply in this species and that infected mosquitoes were capable of transmitting the virus to a susceptible host. In that study, mosquitoes infected via an artificial blood feeding on a mouse skin membrane transmitted ZIKV to a rhesus monkey up to 72 days post mosquito infection, suggesting the high persistence of the virus in the salivary glands [27]. The same year, the experimental infection of a human with ZIKV could not confirm its transmission via *A*. *aegypti* having blood meal on that human [54]. Only 1 study was conducted in the next 50 years, and this study confirmed the ability of *A*. *aegypti* to transmit the virus to a new host in experimental conditions. This observation was made during a comparison between experimental transmission of yellow fever and ZIKV and observing effects on mice previously inoculated intracerebrally with homogenates of infectious mosquitoes [28]. While methods of ZIKV titration were generally based on intracerebral inoculation of the virus in mice, after 2012, the literature describes less-invasive methods such as Vero cell line or plaque assay (S1 Table). The infectivity titers of a virus can be determined by infecting a particular cell line with increasing dilutions of the virus material and determining the highest dilution producing cytopathic effect in 50% of the inoculated cells. In this case, the 50% endpoint dilution is expressed as tissue culture infectious dose_50_ (TCID 50/mL). Plaque assays remain one of the most accurate methods for the direct quantification of infectious virion through the counting of discrete plaques [84] and are the most used method in ZIKV vector competence studies (S1 Table). These methods are generally complemented with molecular techniques such as quantitative real-time (qRT)-PCR that can easily show that viral RNA is present. However, the sole use of this technique indicates the presence of RNA and does not mean that any live virus is present, and the technique needs further testing.

Since 2012, about 30 studies have evaluated *A*. *aegypti* competence for ZIKV transmission in experimental conditions [29–48,50–53,55,85]. Even if it is now well accepted that *A*. *aegypti* is the main vector of ZIKV in urban areas, experimental studies are not entirely consistent regarding the stages of infection, the spread of the virus to the salivary glands, and the incubation period in the mosquito’s body.

The extrinsic incubation period for ZIKV in *A*. *aegypti* varies between these different studies, as do the infection rate, the dissemination rate (with first detection of the virus in the salivary glands between 6 and 14 days post infection [DPI]), and the transmission rate and efficiency. These disparities might be explained by the way these studies were conducted. Several studies have shown that feeding on a viremic animal tends to produce significantly higher infection, dissemination, and transmission rates than feeding on an artificial blood meal through a membrane [34,35,44,70]. Similarly, using nonfrozen, freshly grown virus tends to produce significantly higher infection (and dissemination and transmission) rates than feeding on an artificial blood meal made with frozen stock virus [39,47]. In addition, the origin—African or Asian—of the ZIKV strain, as well as the origin of the population of mosquitoes, may impact the infection, dissemination, and transmission rates [39,44,48]. However, even if the coupling mosquito virus strain is important, the origin of the virus is not sufficient to explain the differences observed in the vector competence studies. Some conflicting results concerning viral transmission were also described. A study on a Senegalese *A*. *aegypti* population described a high transmission rate of about 88% from 7 DPI [28], but mosquitoes were inoculated with ZIKV, a method which cannot be compared with standard vector competence studies. An *A*. *aegypti* population from Singapore orally infected with ZIKV via an artificial blood meal resulted in salivary gland infection rates of 100% at 10 DPI [29]. High concentrations of ZIKV in the blood meal provided to *A*. *aegypti* mosquitoes from Mexico exhibited high transmission efficiency [47]. Brazilian, Australian, and Chinese populations of *A*. *aegypti* infected with ZIKV revealed high transmission efficiency, up to 90% at 14 DPI [34,36,40,46,51]. A laboratory strain of *A*. *aegypti* coinfected with both ZIKV and CHIKV also showed high transmission efficiency for ZIKV [53].

Nevertheless, to our knowledge, these are the only studies providing such a high transmission efficiency for this species, contrasting with the generally low or moderate transmission efficiency observed in various strains of *A*. *aegypti*, even those highly susceptible to ZIKV [30–33,35,38,39,42,44,55]. The methods used in the latter competence studies are overall in agreement regarding the origin of the virus (mainly the Asian strain), the viremia of the blood meal, and the mode of administration (mainly artificial blood feeding through membrane). Methods to evaluate ZIKV transmission are generally based on detection or titration of the virus (mainly plaque or TCID assays on saliva expectorates, complemented by qRT-PCR; S1 Table). In 2017, a study highlighted another approach that may be used for “natural transmission,” with a successful transmission of ZIKV by an infectious *A*. *aegypti* bite to a live mouse [52]. However, the engorgement methods (mosquitoes feeding on viremic animal or with an artificial blood meal, the type of membranes), the preparation of the blood meal (virus freshly grown or from a frozen stock), the different virus primers used for detection, the microbiome, the virome, and the origin of the mosquito population are all factors that may lead to the observed conflicting results and require further investigation (S1 Table). For example, significant variation in competency for ZIKV transmission among *A*. *aegypti* mosquito populations from the Americas was highlighted [30,43]. Actually, the genetic diversity of different populations of *Aedes* species may largely explain those differences, as was observed for the transmission rate for several distinct populations of *A*. *aegypti* for DENV [86] and CHIKV [87].

#### A. albopictus

Native to Southeast Asian tropical and subtropical regions, *A*. *albopictus* became well adapted to temperate regions, and its distribution now includes North America and Europe [88]. In Europe, this mosquito was initially found in the area around the Mediterranean Sea, but it is unexpectedly and progressively spreading further north in Europe [89]. This quick spread of the “Asian tiger mosquito” means that tropical arboviruses are becoming a concern for populations in temperate climates. During the severe 2005–2007 CHIKV epidemic reported in the Indian Ocean, *A*. *aegypti* but also *A*. *albopictus* were described as the main vectors of this virus, as was observed on the French island of Réunion, where *A*. *albopictus* was the dominant species [90]. Although *A*. *albopictus* is not suspected to be the main vector of the most recent arbovirus outbreaks in South America, its similarity to *A*. *aegypti* [91], its implication in CHIKV and DENV transmission [92,93], and its distribution in the Americas, Western Europe, and South and East Asia means that this species remains the focus of much attention [94].

The vector competence of *A*. *albopictus* has been well established for CHIKV and DENV [87,93], but ZIKV transmission remained to be clarified at the beginning of the American outbreak because few studies had clearly demonstrated the ability of this species to transmit the virus. The first suspicion of the potential role of this species in ZIKV transmission appeared in Indonesia, where *A*. *albopictus* and *A*. *aegypti* were widely distributed close to some human ZIKV-infected patients [80]. The first evidence of *A*. *albopictus* competence in transmitting ZIKV was provided in 2013 from a mosquito population in Singapore [69]. This study revealed that this species was susceptible to the Ugandan strain of ZIKV, with high dissemination rates and high transmission rates and efficiency. Seven DPI, all infected mosquitoes exhibited ZIKV in their salivary glands, contrasting with the results observed during the ensuing years, when transmission efficiency was much lower [30,32,35,42,46–48]. In these studies, populations of *A*. *albopictus* from Brazil, the US, China, or Italy infected with the Asian strain of ZIKV had overall lower infection, dissemination, and transmission rates than *A*. *aegypti* but were capable of transmitting ZIKV despite a low efficiency. However, Australian *A*. *albopictus* infected with the Asian strain of ZIKV revealed high transmission efficiency [51], which is concordant with an *A*. *albopictus* competence study from Singapore [69].

#### Other *Aedes* species

Although recent studies have mainly focused on the above species, other mosquito species from the genera *Aedes* or *Culex* may have the potential to be good vectors, depending on their geographical distribution. Most of the studies concerning vector competence in other species were conducted after the beginning of the outbreak in the Americas, except for some old studies conducted in the 1950s in Africa. Indeed, *A*. *aegypti* may not alone account for the extent of this epidemic, and some other species coexisting in the same urban areas might potentially be ZIKV vectors. In addition to *A*. *aegypti* and *A*. *albopictus*, 11 *Aedes* species, including sylvatic and urban species, have been tested experimentally for their competence (Table 1; S1 Table), showing that *A*. *luteocephalus* and *A*. *vittatus* from Senegal have the viral genome in their saliva 15 DPI and, consequently, the potential to transmit ZIKV [55]. In the same study, *Aedes unilineatus* had low infection and dissemination rates and was not able to transmit the virus. Similarly, laboratory colonies of *Aedes triseriatus*, a mosquito known to be a vector of La Crosse virus, tolerant of a wide range of temperatures and broadly distributed across North America, was able to be infected by ZIKV but exhibited dissemination and transmission rates of 0% [35]. However, in this study, samples were in too short supply, which could explain this null rate, and further investigation on *A*. *triseriatus* competence for ZIKV is required. Other *Aedes* species, including *Aedes notoscriptus*, *Aedes procax*, and *Aedes vigilax* collected in Australia were also infected by ZIKV with a prototype of the African strain. Although methods were similar to other studies performed with *A*. *aegypti*, these species did not contain virus in the saliva expectorates and, consequently, were not able to transmit ZIKV [38]. However, in another study, Australian *A*. *notoscriptus* and *Aedes camptorhynchus* had a low transmission efficiency, suggesting that even though they are probably unable to sustain large outbreaks, these species could trigger some secondary cases [51]. Other vector competence studies on *Aedes vexans* collected in the northern US revealed its capacity to transmit ZIKV [71,72]. *A*. *vexans* is one of the most abundant mosquito species in the northern US. This aggressive mosquito has a long flight range, feeds primarily on large mammals, and attacks humans both day and night [95]. Its feeding behavior, combined with its ability to transmit ZIKV, could contribute to its role as a potential vector of ZIKV in the Northern Hemisphere. No evidence of ZIKV transmission was highlighted in a population of *A*. *polynesiensis* sampled on Tahiti Island, France, without any viral particle found in the saliva [31], nor in 1 *Aedes taeniorhynchus* strain from the US coast of the Gulf of Mexico that was refractory to ZIKV infection [70]. *A*. *polynesiensis* was nevertheless highly suspected in transmission on the Pacific Islands because of its ability to transmit DENV, CHIKV, and the Ross River virus [96–98].

#### *Culex* species

Despite the lack of evidence that various other mosquito species can transmit ZIKV, the supposition that a genus other than *Aedes*, such as *Culex*, might be competent in transmitting this virus remains strong. For example, *C*. *quinquefasciatus* is one of the most abundant mosquitoes in anthropized tropical and subtropical areas, as is *C*. *pipiens* in anthropized temperate regions. These species have been studied since the beginning of the outbreak in the Americas, and some discrepancies exist concerning their ability to transmit the virus. The absence of, or very low, infection rates, without any subsequent ZIKV transmission, was observed experimentally in *C*. *quinquefasciatus* from Brazil, the US, China, and Australia [24,36,38,39,43,46,48,49,51,70,73,74]. However, 1 study conducted in Brazil revealed that ZIKV was able to replicate in the body of the mosquito [37]. Nevertheless, this experiment is based on ZIKV RNA detection without detection of infectious virus, leading to the need for assessing their result. Meanwhile, Chinese *C*. *quinquefasciatus* was described as a competent vector for ZIKV in experimental conditions [75]. Though controversial, these studies suggest that caution should be taken with regard to the status of C*ulex*. As suggested for *Aedes* species, the method by which the mosquitoes were fed, the different levels of viremia delivered to the mosquitoes, the virome, the microbiome, and the origin of the mosquito population may influence the experimental vector competence of this species.

Evidence for the lack of competence of other *Culex* species was recently provided via populations of *C*. *pipiens* from Italy, the US, Tunisia, and Germany that were experimentally exposed to ZIKV through an artificial blood meal without being infected [33,35,39,42,49,73,74]. Similarly, ZIKV infection could not be detected in *Culex sitiens* and *Culex annulirostris* from Australia [38,51] nor for *Culex torrentium* and *Culex molestus* from Germany [42].

The lack of competence of *Culex* species was reinforced by the absence of infection in a natural population of *C*. *quinquefasciatus* collected during an outbreak of ZIKV in Mexico [58], and in *C*. *quinquefasciatus* collected in the vicinity of suspected cases of ZIKV infection in Rio de Janeiro, Brazil, from June 2015 to May 2016 [59].

Based on their refractoriness to infection and the absence of viral particles in the saliva, it appears that the tested *Culex* species could possess a midgut barrier that corresponds to the site involved in the first stages of viral attachment, penetration, and replication. This was suggested by studies of the viral competence of *C*. *quinquefasciatus* and *C*. *pipiens* in which the mosquito midgut barrier was bypassed by inoculating the virus directly into the hemocoel, but neither dissemination nor transmission was observed [49,73]. On the other hand, this could reflect the general refractoriness of the mosquito species to ZIKV.

#### *Anopheles* species

Only 2 *Anopheles* species have been tested in vector competence studies, *A*. *gambiae* and *Anopheles stephensi* [24]. These 2 species were not able to be infected, suggesting that they do not play a role in ZIKV transmission to humans. Although *Anopheles* mosquitoes are mostly known to transmit parasites, they are also responsible for transmission of O’nyong-nyong virus, which is closely related to CHIKV [99].

There is, consequently, a need to conduct field studies to identify the largest number of species from various genera that are potential vectors of ZIKV, as well as laboratory studies to confirm the lack of competence of these species. Moreover, the distinction between vectorial capacity and vector competence must be observed with caution. Vectorial capacity refers to all of the environmental, behavioral, cellular, and biochemical factors that may have an influence on the association between a vector, the pathogen transmitted by the vector, and the vertebrate host to which the pathogen is transmitted [100]. Vectorial capacity is essentially determined by both environmental and behavioral factors. For example, a particular mosquito species might be genetically and biochemically compatible for the complete development of a particular pathogen (i.e., vector competent for this pathogen), but if this species does not coexist temporally and spatially with a vertebrate host that harbors the pathogen, or if the preferred blood source for this species does not include that vertebrate, this mosquito would not be a suitable vector for this pathogen [100]. Therefore, a mosquito species that has a high vector competence would not automatically imply that this species is a vector of epidemiological importance. For these reasons, *A*. *aegypti* remains the species most likely to be responsible for the spread of the virus in the Americas.

### *A*. *aegypti* and the outbreak in the Americas

#### Low vector competence but high vectorial capacity

Even if in experimental conditions *A*. *aegypti* and *A*. *albopictus* show little vector competence, this does not reflect their potential to cause epidemics, driven not only by their vector capacity (i.e., their large populations and host feeding preferences and frequencies) but also by human parameters. Indeed, one of the hypotheses explaining the drastic spread and virulence of the outbreak concerns the origin of the virus. In the Americas, the virus has been described to belong to the Asian genotype and is closely related to the strain that circulated in French Polynesia in 2013 [101]. Moreover, vector competence assays with the strain circulating in the Americas did not show high infectivity for *A*. *aegypti*, suggesting that the sole origin of the virus could not explain the differences between the outbreak in the Americas and previous outbreaks [39]. The limited diagnostic capabilities in Africa and Asia (i.e., the absence of field research in areas where other arboviral infections were present), combined with a relative immune population, might partly explain why it became a threat in the Americas with a naïve human population. Increased global travel, uncontrolled urbanization associated with areas where the possibility of maintaining proper hygiene is poor, and populations with limited access to water—requiring them to store water, leading to increased mosquito breeding sites—might explain the extent of this epidemic.

*Aedes* eggs are desiccation-resistant and can consequently survive for long periods, leading to the potential persistence of arbovirus in the eggs. Like for other flaviviruses such as DENV [102] or yellow fever [103], vertical transmission of ZIKV in *A*. *aegypti* and *A*. *albopictus* was demonstrated [45,104,105]. The filial infection rate (FIR) in *A*. *aegypti* mosquitoes injected intrathoracically with ZIKV was 1/290 [104]. In another study, *A*. *aegypti* and *A*. *albopictus* receiving a viremic blood meal had a FIR of 1/84 [105]. This value is not entirely consistent with the previous study and appears to be high compared with the FIR observed for other flaviviruses. ZIKV seems to have a great capacity to be transmitted vertically by *A*. *aegypti* and *A*. *albopictus*, and it has been observed that this vertical transmission may be different depending on the origin of the mosquito. It seems that vertical transmission may play a role in the propagation and maintenance of ZIKV, but the impact of this transmission appears to be negligible compared with horizontal transmission [105].

Moreover, despite the absence of evidence, some studies hypothesized that a link exists between climatic events such as El Niño and the spread of ZIKV from Brazil to North America [106–109]. El Niño leads to extreme temperatures in northern South America, which might enhance the development of *A*. *aegypti*. Moreover, higher temperatures can increase the habitat of this tropical mosquito and might have an influence on the physiology of the mosquito through higher biting rates, lower mortality, and smaller extrinsic incubation periods. However, the link between ZIKV outbreak in the Americas and this climatic event should be taken with caution insofar as the major CHIKV outbreak in the same area, which is transmitted by the same vector—*A*. *aegypti*—occurred in 2014, which was not an El Niño year. Even if *A*. *aegypti* does not have a good vector competence, the main reason for its good vectorial capacity relies on the fact that most of the individuals mainly feed on humans with multiple bites in a single gonotrophic cycle [110], which contrasts with the behavior of other mosquito species found during the 2015 outbreak. Moreover, locally, cases of ZIKV were all acquired in areas where *A*. *aegypti* is present, suggesting that if other species were involved in any significant extent, then there would have been cases in other areas.

#### Strategies for mosquito management

The project of eradication of the *A*. *aegypti* mosquito began in the first half of the 20th century, after it had been established that this mosquito transmitted the urban yellow fever. Sanitation reform, in particular getting rid of stagnant water, where this mosquito lays its eggs, was the most effective way to eradicate *A*. *aegypti*, in combination with the use of insecticides to fight adult mosquitoes [111]. In 1934, Brazil had managed to eradicate the mosquito in several cities in its northeast, and the country launched efforts to do so nationwide. In 1942, Brazil was declared to be free of *A*. *aegypti*, through a combination of public education and fumigation, mainly with the organochlorine dichlorodiphenyltrichloroethane (DDT). Five years later, several South American countries, in association with the Pan American Sanitary Organization, planned on wiping out *A*. *aegypti* across the continent [111]. In 1962, after years of indoor residual spraying of high doses of DDT, military-grade organization, and funding to train personnel and buy equipment, the eradication of *A*. *aegypti* became successful in 18 countries of the Americas plus several islands of the West Indies. As these efforts succeeded, *A*. *aegypti* control lost political importance, and attention and funding declined [111]. The failure to maintain this success was and is still now complicated by population increase, extensive and sprawling urbanization, and the lack of municipal infrastructure such as piped water. This failure could mainly explain the regular invasions of DENV, CHIKV, and ZIKV in South America.

Today, the use of insecticides is one of the major components in the global strategy to control mosquito-associated diseases. Indeed, to fight populations of mosquitoes, the available tools include insecticide spatial spraying and reducing the production of larvae. Other methods are used around the world, depending on the legislation of the country, such as introducing *Wolbachia* intracellular bacteria into the bodies of *A*. *aegypti* individuals with the intent to shorten the lifespan of female mosquitoes [112]. Another method carried out in Brazil was the use of transgenic *A*. *aegypti*, which expresses a self-limiting transgene in order to prevent larvae from developing to adulthood [113]. In the absence of controls over these methods, which are highly controversial and also depend on the country’s legislation, the main global mosquito management strategy remains removing breeding sites and the use of insecticides. The use of insecticides always implies a delicate balance between, on the one hand, their efficacy and the resistance of the target populations and, on the other hand, the demonstrated or supposed toxicity for humans and the environment. In South America, the main target of the antivectorial fight to prevent ZIKV spread was the urban mosquito *A*. *aegypti*.

However, if ZIKV had a sylvatic cycle in South America, it would involve other mosquito species that would not be compatible with the current vector control. In Africa and Asia, sylvatic cycles have been described with ZIKV, involving many species of animals in which the virus or antibodies have been isolated, such as monkeys, rodents, bats, orangutans, and carabaos [114]. In the Americas, studies on ZIKV in vertebral hosts are scarce, but the remarkable diversity of Latin American wildlife species provides a potential to establish a sylvatic ZIKV cycle, along with more than 200 mosquito species, which needs to be explored and surveyed [115]. A sylvatic cycle of ZIKV would make its elimination almost impossible, and the use of insecticides remains focused on the urban mosquito *A*. *aegypti*. However, the widespread use of these chemical compounds around the world has led to resistant phenotypes in vector populations [116–118]. These resistant phenotypes are due to a combination of multiple and complex mechanisms, such as the greater metabolic detoxification of insecticides before they reach the nervous system [119–121] and the decreased sensitivity of the targeted proteins through mutations of the neural targets of the insecticides [121]. A question remains to be elucidated; namely, can insecticide resistance interact with pathogen transmission and particularly for ZIKV in mosquitoes? First, insecticide resistance may have a direct impact on ZIKV outbreaks by making it more difficult to satisfactorily reduce mosquito populations [122]. Secondly, the molecular mechanisms involved in detoxification or in gene response to insecticide exposure such as immune response may interact with signaling pathways involved in response to viral infection, as observed in *A*. *gambiae* with the parasite *Plasmodium falciparum*, in which positive or negative interactions were observed [123–125]. The number of mosquitoes in a population and the lifespan of resistant insects may be higher in regions where insecticides are used [126,127]. Consequently, the observed high resistance rates in the *A*. *aegypti* population of South America may have enhanced the transmission and spread of ZIKV.

## Conclusion

Despite several years of entomological investigations and the discovery of many mosquito species naturally infected by ZIKV, recent advances revealed that *A*. *aegypti* may be the major vector driving recent epidemics, while other *Aedes* species may contribute to the sylvatic transmission cycle of ZIKV. Since the beginning of the ZIKV outbreak in the Americas, the number of studies has soared, and it has been shown that the epidemic resulted from a combination of several factors, including highly anthropophilic mosquito behavior, environment parameters, and human factors such as population increase, urbanization, and the failure to provide municipal services such as piped water. Although our knowledge increases concerning the vector competence of *Aedes* and C*ulex* species, there is still a need to explore which other species could be competent for ZIKV. At a time when data on sexual transmission in humans are oriented towards specific public health measures, including state recommendations on sexuality and the procreation of individuals living in an epidemic area, further research on mosquito infection and transmission is fundamental.

## Supporting information

S1 Table Comparison of vector competence studies of ZIKV. Virus titres calculated by the method of Reed and Muench are expressed as the reciprocal of the log_10_ dilution, which killed 50% of the mice inoculated. The TCID_50_ test quantifies the amount of virus required to produce cytopathic effect in 50% of inoculated tissue culture cell. PFU is a measure of the number of particles capable of forming plaques per unit volume. FFU is the unit of a variant of the plaque assay, the FFA based on immunostaining techniques. The infection rate corresponds to the proportion of mosquitoes with virus-infected bodies among those tested. The dissemination rate corresponds to the proportion of mosquitoes with virus-infected legs among those infected. The transmission rate corresponds to the proportion of mosquitoes with infectious saliva among those infected. The transmission efficiency corresponds to the proportion of mosquitoes with infectious saliva among those tested. In the table, high infection, dissemination, or transmission were arbitrarily chosen as values greater than 60% among the tested mosquitoes, a moderate value between 40% and 60%, and a low value less than 40%. AP-61, *Aedes pseudoscutellaris* 61; CHIKV, chikungunya virus; DPI, day post infection; FFA, focus forming assay; FFU, focus forming unit; NA, not available; PFU, plaque forming unit; TCID, tissue culture infectious dose; ZIKV, Zika virus.
(DOCX)
