# Supplementary material for: Zika virus: An updated review of competent or naturally infected mosquitoes
Source: PLoS Negl Trop Dis. 2017 Nov 16;11(11):e0005933. doi: 10.1371/journal.pntd.0005933 (PMC5690600; doi:10.1371/journal.pntd.0005933)
Supplement: S1 Table — Virus titres calculated by the method of Reed and Muench are expressed as the reciprocal of the log10 dilution, which killed 50% of the mice inoculated. The TCID50 test quantifies the amount of virus required to produce cytopathic effect in 50% of inoculated tissue culture cell. PFU is a measure of the number of particles capable of forming plaques per unit volume. FFU is the unit of a variant of the plaque assay, the FFA based on immunostaining techniques. The infection rate corresponds to the proportion of mosquitoes with virus-infected bodies among those tested. The dissemination rate corresponds to the proportion of mosquitoes with virus-infected legs among those infected. The transmission rate corresponds to the proportion of mosquitoes with infectious saliva among those infected. The transmission efficiency corresponds to the proportion of mosquitoes with infectious saliva among those tested. In the table, high infection, dissemination, or transmission were arbitrarily chosen as values greater than 60% among the tested mosquitoes, a moderate value between 40% and 60%, and a low value less than 40%. AP-61, Aedes pseudoscutellaris 61; CHIKV, chikungunya virus; DPI, day post infection; FFA, focus forming assay; FFU, focus forming unit; NA, not available; PFU, plaque forming unit; TCID, tissue culture infectious dose; ZIKV, Zika virus. (DOCX) [file pntd.0005933.s001.docx]

| **Reference** | **Mosquito species** | **Mosquito origin** | **Mosquitoes tested/ Time point** | **Age of bloodfed females in days** | **Method of infection** | **Final viremia into inoculate** | **ZIKV strain** | **Transmission assay** | **Method of ZIKV detection and titration** | **Observation** |
| --- | --- | --- | --- | --- | --- | --- | --- | --- | --- | --- |
| Boorman & Porterfield 1956 | *Ae. aegypti* | Nigeria | NC | 5 | Artificial blood feeding on mouse skin membrane | 10^6.7^ mouse LD50 | Uganda | Engorgement on mice and homogenates of mosquitoes inoculate intracerebrally to mice | Titration in mice (Reed and Muench, 1938^*^) | - Detection of the virus from day 20 to 60.  - Transmission of ZIKV efficient trough bites of three infected mosquitoes (72 DPI) on a rhesus monkey. |
| Bearcroft 1956 | *Ae. aegypti* | India | NC | 5 and 8 | Blood feeding on ZIKV-infected Human | NC | Eastern Nigeria | Engorgement on mice and homogenates of mosquitoes inoculate intracerebrally to mice | Titration in mice (Reed and Muench, 1938), and haemagglutination-inhibition test | - No transmission of ZIKV from *Ae. aegypti* to mice. |
| Cornet et al. 1979 | *Ae. aegypti* | Senegal | 30 | NC | Intrathoracic inoculation | NC | ArD 24280 from *Ae. luteocephalus* | Engorgement on mice and homogenates of mosquitoes inoculate intracerebrally to mice | Titration in mice and complement fixation test | - High transmission rate from day 7 (88%) to day 30 (79%) post infection.  - The study compares the transmission between ZIKV and YFV. |
| Li et al. 2012 | *Ae. aegypti* | Singapore | 8 | 5 to 7 | Artificial blood feeding | 7 log_10_ TCID_50_/mL | MR766 from Uganda | Presence of ZIKV in salivary glands | Titration by TCID_50_ assay | - Detection of ZIKV in salivary glands from 4 DPI with infection rate of 100% at 6 DPI.  - High dissemination rate (100% at 10 DPI). |
| Wong et al. 2013 | *Ae. albopictus* | Singapore | 12 | 5 to 7 | Artificial blood feeding | 7.5 log_10_ TCID_50_/mL | MR766 from Uganda | Presence of ZIKV in saliva | Titration by TCID_50_ assay and qRT-PCR | - High infection rate (100% from 3 to 14 DPI).  - High dissemination rate (100% at 7 DPI).  - High transmission efficiency (100% at 10 DPI). |
| Diagne et al. 2015 | *Ae. aegypti* | Dakar/Kedougou, Senegal | NC | 7 | Artificial blood feeding | 10^7^ PFU/mL (Ugandan strain) | - ArD 128000, ArD 132912, ArD 157995, ArD 165522 isolated from mosquitoes from Senegal  - HD787881 isolated from human blood from Senegal  - MR766 from Uganda | Presence of ZIKV in saliva | qRT-PCR | - Moderate infection rate whatever the viral strain.  - Low dissemination rate.  - No transmission (0% at each point tested). |
|  | *Ae. unilineatus* | Kedougou, Senegal |  |  |  | 2x10^7^ PFU/mL (Ugandan strain) |  |  |  | - Low infection rate.  - Low dissemination.  - No transmission (0% at each point tested). |
|  | *Ae. vittatus* | Kedougou, Senegal |  |  |  | 3.3x10^7^ PFU/mL (Ugandan strain) |  |  |  | - Low infection rate.  - Low dissemination.  - Low transmission efficiency. |
|  | *Ae. luteocephalus* | Kedougou, Senegal |  |  |  | 2x10^7^ PFU/mL (Ugandan strain) |  |  |  | - High infection rate.  - High dissemination rate.  - Low transmission efficiency. |
| Chouin-Carneiro et al. 2016 | *Ae. aegypti* | French Guiana, France | 25-30 | 7 | Artificial blood feeding | 10^7^ TCID_50_/mL | NC-2014-5132 isolated from human blood from New Caledonia | Presence of ZIKV in saliva | Titration by plaque assay and TCID_50_ assay. | - *Ae. albopictus* from Florida more susceptible to ZIKV infection than *Ae. albopictus* from Rio de Janeiro.  - Transmission of 0% for *Ae. aegypti* and *Ae. albopictus* at 4 and 7 DPI whatever the population considered.  -Low transmission efficiency of *Ae. aegypti* from Brazil and *Ae. albopictus* from USA at 14 DPI. |
|  | *Ae. aegypti* | Guadeloupe, France |  |  |  |  |  |  |  |  |
|  | *Ae. aegypti* | Martinique, France |  |  |  |  |  |  |  |  |
|  | *Ae. aegypti* | Orlando, USA |  |  |  |  |  |  |  |  |
|  | *Ae. aegypti* | Rio de Janeiro, Brazil |  |  |  |  |  |  |  |  |
|  | *Ae. albopictus* | Rio de Janeiro, Brazil |  |  |  |  |  |  |  |  |
|  | *Ae. albopictus* | Florida, USA |  |  |  |  |  |  |  |  |
| Di Luca et al. 2016 | *Ae. aegypti* | Mexico | 10 | 10 | Artificial blood feeding | 6.46 log_10_ PFU/mL | ZIKV isolated from a patient returning from French Polynesia in 2013 | Presence of ZIKV in saliva | Titration by plaque assay and qRT-PCR | - Transmission observed at 4 to 21 DPI for *Ae. aegypti* and at 11 to 14 DPI for *Ae. albopictus.*  - *Ae aegypti* more competent than *Ae. albopictus.* |
|  | *Ae. albopictus* | Calabria, Italia |  |  |  |  |  |  |  |  |
| Aliota et al. 2016a | *Ae. aegypti* | Medellin, Colombia | 20-30 | 3 to 6 | Blood feeding on ZIKV-infected mice | 6.02 log_10_ PFU/mL | PRVABC59 isolated from human blood from Puerto Rico | Presence of ZIKV in saliva | Titration by plaque assay and qRT-PCR | - Infection of *Ae. aegypti* from 4 DPI with extrinsic incubation period from 7 to 14 DPI.  - Mosquitoes feeding on ZIKV-infected mice with high viremia and with artificial blood feeding start to have saliva infected from 7 DPI.  - Mosquitoes feeding on ZIKV-infected mice with low viremia start to have saliva infected from 14 DPI. |
|  |  |  |  |  | Blood feeding on ZIKV-infected mice | 4.74 log_10_ PFU/mL |  |  |  |  |
|  |  |  |  |  | Artificial blood feeding | 8.0 log_10_ PFU/mL |  |  |  |  |
|  | *Ae. aegypti* | Cross between males from Colombia and wMel-infected female | 20-30 |  | Blood feeding on ZIKV-infected mice | 6.02 log_10_ PFU/mL |  |  |  | - *Ae. aegypti* wMel feeding on ZIKV-infected mice with high viremia have a significant reduction in ZIKV infection and dissemination compared to control, but no transmission was observed.  - *Ae. aegypti* wMel feeding on ZIKV-infected mice with low viremia and with artificial blood are not capable of disseminating and transmitting ZIKV. |
|  |  |  |  |  | Blood feeding on ZIKV-infected mice | 4.74 log_10_ PFU/mL |  |  |  |  |
|  |  |  |  |  | Artificial blood feeding | 8.0 log_10_ PFU/mL |  |  |  |  |
| Aliota et al. 2016b | *Ae. aegypti* | University of Wisconsin-Madison, USA | NC | 3 to 6 | Blood feeding on ZIKV-infected mice | 6.02 log_10_ PFU/mL | PRVABC59 isolated from human blood from Puerto Rico | Presence of ZIKV in saliva | Titration by plaque assay | - *Ae. aegypti* is infected and can disseminate and transmit ZIKV at 14 DPI. |
|  |  |  | NC |  |  | 4.74 log_10_ PFU/mL |  |  |  |  |
|  |  |  | 17 |  |  | 6.83 log_10_ PFU/mL |  |  |  |  |
|  | *Ae. albopictus* |  | 9 |  | Blood feeding on ZIKV-infected mice | 6.02 log_10_ PFU/mL |  |  |  | - High dissemination but low transmission efficiency of *Ae. albopictus* at 14 DPI. |
|  |  |  | 9 |  |  | 4.74 log_10_ PFU/mL |  |  |  |  |
|  |  |  | NC |  |  | 6.83 log_10_ PFU/mL |  |  |  |  |
|  | *Ae. triseriatus* |  | NC |  | Blood feeding on ZIKV-infected mice | 6.02 log_10_ PFU/mL |  |  |  | *- Ae. triseriatus* is susceptible to ZIKV infection when exposed to mice with the highest viremia concentration, but no dissemination nor transmission was observed at 14 DPI. |
|  |  |  | 20 |  |  | 4.74 log_10_ PFU/mL |  |  |  |  |
|  |  |  | 13 |  |  | 6.83 log_10_ PFU/mL |  |  |  |  |
|  | *Cx. pipiens* |  | 20 |  | Blood feeding on ZIKV-infected mice | 6.02 log_10_ PFU/mL |  |  |  | -No infection, nor dissemination, nor transmission of ZIKV in *Cx. pipens* at 14 DPI. |
|  |  |  | 10 |  |  | 4.74 log_10_ PFU/mL |  |  |  |  |
|  |  |  | 30 |  |  | 6.83 log_10_ PFU/mL |  |  |  |  |
| Boccolini et al. 2016 | *Ae. aegypti* | Mexico | 8-12 | 10 | Artificial blood feeding | 6.46 log_10_ PFU/mL | H/PF/2013 isolated from human blood from French Polynesia | Presence of ZIKV in saliva | Titration by plaque assay and qRT-PCR | - High infection rate but low dissemination rate and transmission efficiency of ZIKV. |
|  | *Cx. pipiens* | Rome, Italia | 10 |  |  |  |  |  |  | - Short persistence of the virus in the mosquito’s body.  - No dissemination, nor transmission of ZIKV. |
| Amraoui et al. 2016 | *Cx. quinquefasciatus* | California, USA | 40-48 | 7 | Artificial blood feeding | 10^7.2^ PFU/mL | NC-2014-5132 isolated from human blood from New Caledonia | Presence of ZIKV in saliva | Titration by plaque assay | - Low infection rate.  - Very low dissemination at 14 and 21 DPI.  - No transmission. |
|  |  |  | 21-24 |  | Intrathoracic inoculation | 2530 PFU |  |  |  | - Very low dissemination at 3 DPI.  - No transmission. |
|  | *Cx. pipiens* | Tabarka, Tunisia | 40-48 |  | Artificial blood feeding | 10^7.2^ PFU/mL |  |  |  | - No infection.  - No dissemination.  - No transmission. |
|  |  |  | 21-24 |  | Intrathoracic inoculation | 2530 PFU |  |  |  | - Very low dissemination at 7 and 14 DPI.  - No transmission. |
| Fernandes et al. 2016 | *Ae. aegypti* | 2 districts of Rio de Janeiro, Brazil | 4-30 | 5 to 7 | Artificial blood feeding | 10^6^ PFU/mL | Rio-U1 isolated from human urine in Rio de Janeiro | Presence of ZIKV in saliva | Titration by plaque assay and qRT-PCR | - High infection, dissemination and transmission rates for the 2 populations of *Ae. aegypti* and the 2 strains of ZIKV. |
|  |  |  |  |  |  |  | Rio-S1 isolated from human saliva in Rio de Janeiro |  |  |  |
|  | *Cx. quinquefasciatus* | 4 districts of Rio de Janeiro, Brazil |  |  |  |  | Rio-U1 isolated from human urine in Rio de Janeiro |  |  | - Very low infection.  - No transmission for all the populations tested. |
|  |  |  |  |  |  |  | Rio-S1 isolated from human saliva in Rio de Janeiro |  |  |  |
| Guo et al. 2016 | *Cx. quinquefasciatus* | Hainan, China | 10 | 7 | Artificial blood feeding | 3x10^5^ PFU/mL | KU866423 isolated from human blood from Samoa | Presence of ZIKV in saliva and engorgement on mice | Titration by plaque assay and qRT-PCR | - Infection from 2 to 18 DPI.  - Dissemination at 6 DPI.  - Transmission observed at 8 and 12 DPI.  - Ten days post engorgement on mice, 89% of the mice have viral RNA in their brain. |
| Guedes et al. 2016 | *Ae. aegypti* | RecLab, Brazil | 4-15 | 7 to 10 | Artificial blood feeding | 10^6^ PFU/mL | BRPE243/2015 isolated from human serum in Brazil | Presence of ZIKV in salivary glands and saliva | Titration by plaque assay and qRT-PCR | - Transmission from 9 to 12 DPI for *Ae. aegypti* and *Cx. quinquefasciatus* whatever the viral dose delivered.  - Both species are susceptible to ZIKV infection, dissemination and transmission. |
|  |  |  |  |  |  | 10^4^ PFU/mL |  |  |  |  |
|  | *Ae. aegypti* | Pernambuco, Brazil |  |  |  | 10^6^ PFU/mL |  |  |  |  |
|  |  |  |  |  |  | 10^4^ PFU/mL |  |  |  |  |
|  | *Cx. quinquefasciatus* | CqSLab, Brazil |  |  |  | 10^6^ PFU/mL |  |  |  |  |
|  |  |  |  |  |  | 10^4^ PFU/mL |  |  |  |  |
| Huang et al. 2016 | *Cx. pipiens* | California, USA | 27-34 | 8 to 10 | Artificial blood feeding | 6.52 log_10_ TCID_50_/mL | NC | NC | Titration by TCID_50_ assay and qRT-PCR | - No infection.  - No dissemination. |
|  | *Cx. pipiens* | New Jersey, USA | 20 |  |  | 7.52 log_10_ TCID_50_/mL |  |  |  | - No infection.  - No dissemination. |
|  | *Cx. quinquefasciatus* | Florida, USA | 30-33 |  |  | 6.95 log_10_ TCID_50_/mL |  |  |  | - No infection.  - No dissemination. |
| Hall-Mendelin et al. 2016 | *Ae. aegypti* | Queensland, Australia | 25-30 | 3 to 7 | Artificial blood feeding | 10^6.7^ TCID_50_/mL and 10-fold serial dilutions | MR766 from Uganda | Presence of ZIKV in saliva | Titration by TCID_50_ assay and qRT-PCR | - Moderate infection at 5, 7, 10 and 14 DPI (~50%).  - Low dissemination.  - Low transmission efficiency at 10 and 14 DPI. |
|  | *Ae. procax* |  | 6 |  |  | 10^6.7^ TCID_50_/mL |  |  |  | - Low infection.  - Very low dissemination.  - No transmission. |
|  | *Ae. vigilax* |  | 30 |  |  | 10^6.7^ TCID_50_/mL |  |  |  | - High infection.  - Low dissemination.  - No transmission. |
|  | *Ae. notoscriptus* |  | 25-30 |  |  | 10^6.7^ TCID_50_/mL and 10-fold serial dilutions |  |  |  | - High infection.  - Low dissemination.  - No transmission. |
|  | *Cx. quinquefasciatus* |  | 30 |  |  | 10^6.7^ TCID_50_/mL |  |  |  | - Very low infection rate.  - No dissemination.  - No transmission. |
|  | *Cx. annulirostris* |  | 30 |  |  | 10^6.7^ TCID_50_/mL |  |  |  | - No infection.  - No dissemination.  - No transmission. |
|  | *Cx. sitiens* |  | 11 |  |  | 10^6.7^ TCID_50_/mL |  |  |  | - No infection.  - No dissemination.  - No transmission. |
| Weger-Lucarelli et al. 2016 | *Ae. aegypti* | Poza Rica, Mexico | 45-48 | 5 to 7 | Artificial blood feeding | 2x10^6^ PFU/mL | PRVABC59 isolated from human blood in Puerto Rico (fresh virus) | Presence of ZIKV in saliva | Titration by plaque assay | - PRVABC59 (Asian strain) is less infectious than the two other strains.  - Long freezing of ZIKV strain lead to less of infectivity compared to fresh or short-term freezing ZIKV.  - No loss of infectivity according to the number of passages on cell lines.  - High infection.  - Moderate dissemination.  - Moderate transmission efficiency |
|  |  |  | 141-144 |  |  | 1.6x10^7^ PFU/mL | PRVABC59 isolated from human blood in Puerto Rico (frozen virus) |  |  |  |
|  |  |  | 48 |  |  | 1.6x10^7^ PFU/mL | MR766 from Uganda |  |  |  |
|  |  |  | 48 |  |  | 1.6x10^7^ PFU/mL | 41525 isolated from *Ae. africanus* in Senegal |  |  |  |
|  | *Cx. quinquefasciatus* | Florida, USA | 48 |  |  | 5x10^6^ PFU/mL | PRVABC59 isolated from human blood in Puerto Rico (fresh virus) |  |  | - No infection except for 1 *Cx. quinquefasciatus* at 7 DPI. |
|  |  |  | 48 |  |  | 1.6x10^7^ PFU/mL | PRVABC59 isolated from human blood in Puerto Rico (frozen virus) |  |  |  |
|  | *Cx. tarsalis* | California, USA | 20 |  |  | 5x10^6^ PFU/mL | PRVABC59 isolated from human blood in Puerto Rico (fresh virus) |  |  | - No infection. |
|  | *Cx. pipiens* | Pennsylvania, USA | 48 |  |  | 5x10^6^ PFU/mL | PRVABC59 isolated from human blood in Puerto Rico (fresh virus) |  |  | - No infection. |
| Richard et al. 2016 | *Ae. aegypti* | Tahiti, France | 39-40 | 5 | Artificial blood feeding | 7 log_10_ TCID_50_/mL | PF13/251013-18 isolated from human blood in French Polynesia | Presence of ZIKV in saliva | Titration by TCID_50_ assay and qRT-PCR | - High infection rate  - Moderate to high dissemination between 6 and 21 DPI.  - Low transmission before 14 DPI but high transmission at 21 DPI. |
|  | *Ae. polynesiensis* | Tahiti, France | 66-95 |  |  |  |  |  |  | - Low infection at 6, 9 and 14 DPI.  - Low dissemination at 9 and 14 DPI.  - No transmission. |
| Dutra et al. 2016 | *Ae. aegypti* | Rio de Janeiro, Brazil | 20 | 4 | Artificial blood feeding | 5x10^6^ PFU/mL | BRPE243/2015 from human serum in Brazil | Presence of ZIKV in saliva and intrathoracic injection of saliva collected from ZIKV-infected mosquitoes | Titration by plaque assay and qRT-PCR | - High infection for *Ae. aegypti* at 14 DPI for all ZIKV strains.  - High dissemination for *Ae. aegypti* at 14 DPI for all ZIKV strains.  - High transmission efficiency for *Ae. aegypti* infected with BRPE ZIKV strain.  - Low infection, and dissemination rates for *w*Mel*-*infected *Ae. aegypti.*  *-* Low transmission efficiency for *w*Mel-infected *Ae. aegypti* with BRPE ZIKV strain.  - Prevalence of ZIKV infection reduced among *Wolbachia*-infected *Ae. aegypti.*  - Injection of infectious saliva into ZIKV-uninfected *Ae. aegypti* or *wMel-infected Ae. aegypti* leads to infection of all *Ae. aegypti* and none of the *w*Mel-infected *Ae. aegypti.* |
|  |  |  | 20 |  |  | 8.7x10^3^ PFU/mL | SPH/2015 from human serum in Brazil |  |  |  |
|  | *Ae. aegypti* | wMel-infected laboratory strain | 20 |  |  | 5x10^6^ PFU/mL | BRPE243/2015 from human serum in Brazil |  |  |  |
|  |  |  | 20 |  |  | 8.7x10^3^ PFU/mL | SPH/2015 from human serum in Brazil |  |  |  |
| Hart et al. 2017 | *Cx. quinquefasciatus* | Gulf Coast, USA | 20 | NC | Artificial blood feeding | 6 log_10_ FFU/mL | MEX 1-44 isolated from *Ae. aegypti* in Mexico | Presence of ZIKV in saliva | Titration by focus forming assay | - No infection.  - No dissemination.  - No transmission. |
|  |  |  | 20 | NC |  | 4 log_10_, 5 log_10_ and 6 log_10_ FFU/mL | MEX 1-7 isolated from *Ae. aegypti* in Mexico |  |  |  |
|  |  |  | 20 | NC |  | 4 log_10_, 5 log_10_ and 6 log_10_ FFU/mL | 41525 isolated from *Ae. africanus* in Senegal |  |  |  |
|  |  |  | 20 | NC |  | 4 log_10_, 5 log_10_ and 6 log_10_ FFU/mL | FSS 13025 isolated from a human in Cambodia in 2010 |  |  |  |
|  | *Cx. quinquefasciatus* | Houston, USA | 5 | NC | Blood feeding on ZIKV-infected mice | 4 log_10_, 6 log_10_ and 7 log_10_ FFU/mL | FSS 13025 isolated from a human in Cambodia in 2010 |  |  |  |
|  |  |  | 26 | NC |  | 6 log_10_ FFU/mL | MEX 1-7 isolated from *Ae. aegypti* in Mexico |  |  |  |
|  |  |  | 21 | NC |  | 7 log_10_ FFU/mL | PRVABC59 isolated from human blood in Puerto Rico |  |  |  |
|  | *Ae. taeniorhynchus* | Gulf Coast, USA | 20 | NC | Artificial blood feeding | 6 log_10_ FFU/mL | MEX 1-44 isolated from *Ae. aegypti* in Mexico |  |  |  |
| Costa-da-Silva. 2017 | *Ae. aegypti* | Rockfeller, lab strain | 20 | 7 to 9 | Artificial blood feeding | 2.2x10^6^ PFU/mL | ZIKV^BR^ from human case in Brazil | Presence of ZIKV in saliva and intrathoracic injection of saliva collected from ZIKV-infected mosquitoes | Titration by qRT-PCR | -High infection (95%), and dissemination rates (100%) at 14 DPI.  -Low transmission rate (36.8%) at 14 DPI. |
|  |  | Rexville, lab strain | 20 |  |  |  |  |  |  | -High infection (95%), and dissemination rates (95%) at 14 DPI.  -Low transmission rate (5.3%) at 14 DPI. |
|  |  | Higgs white eyes, lab strain | 20 |  |  |  |  |  |  | -High infection (65%), and dissemination rates (100%) at 14 DPI.  -Low transmission rate (30.8%) at 14 DPI. |
|  |  |  | 20-25 | 5 to 7 | Intrathoracic inoculation with infectious saliva collected at 7 and 14 DPI from one of the 3 *Ae. aegypti* lab strains | NC |  |  |  | Confirmation of infectivity of the saliva from Rockfeller, Rexville or Higgs white eyes strains collected at 7 DPI or 14 DPI |
| Heitmann et al. 2017 | *Ae. aegypti* | Bayer company, lab strain | 50-63 | 4 to 14 | Artificial blood feeding | 10^7^ PFU/mL | ZIKV_FB-GWUH-2016 isolated in a fetal brain | Presence of ZIKV in saliva | Titration by qRT-PCR | High infection (49-72 %) and low transmission rates (45-31 %) at 14 and 21 DPI. |
|  | *Ae. albopictus* | Freiburg, Germany | 31-34 |  |  |  |  |  |  | High infection (53-65 %) and low transmission rates (20-33 %) at 14 and 21 DPI. |
|  | *Ae. albopictus* | Calabria, Italy | 29-31 |  |  |  |  |  |  | High infection (52-71 %) and low transmission rates (13-18 %) at 14 and 21 DPI. |
|  | *Cx. molestus* | Germany, lab strain | 29-38 |  |  |  |  |  |  | -Low infection rate (24-32 %) at 14 and 21 DPI.  -No transmission. |
|  | *Cx. pipiens* | Hamburg, Germany | 35-37 |  |  |  |  |  |  | -Low infection rate (0-8 %) at 14 and 21 DPI.  -No transmission. |
|  | *Cx. torrentium* | Hamburg, Germany | 34-36 |  |  |  |  |  |  | -Low infection rate (0-11 %) at 14 and 21 DPI.  -No transmission. |
| Dodson and Rasgon. 2017 | *An. Gambiae* | Pennsylvania, USA | 11-24 | 3 to 5 | Artificial blood feeding | 4.6 log_10_ PFU/mL | MR766 from Uganda | Presence of ZIKV in saliva | Titration by plaque assay | -No infection. |
|  |  |  | 11-19 |  |  | 7 log_10_ PFU/mL |  |  |  |  |
|  | *An. stephensi* | Maryland, USA | 30-35 |  |  | 4.3 log_10_ PFU/mL |  |  |  |  |
|  |  |  | 30-33 |  |  | 7.7 log_10_ PFU/mL |  |  |  |  |
|  | *Cx. quinquefasciatus* | New York, USA | 29-30 |  |  | 7.5 log_10_ PFU/mL |  |  |  |  |
|  |  |  | 26-30 |  |  | 7.3 log_10_ PFU/mL | PRVABC59 isolated from human blood in Puerto Rico |  |  |  |
| Fernandes et al. 2017 | *Ae. aegypti* | Rio de Janeiro, Brazil | 20-39 | 5 to 7 | Artificial blood feeding | 2.3 x 10^6^ PFU/mL | ZIKVPE243 isolated in 2015 from a human in Recife, Brazil | Presence of ZIKV in saliva tested if evidence of dissemination | Titration by plaque assay and qRT-PCR | -High infection rate (65-75% at 7 dpi and 68-100% at 14 dpi)  -High dissemination rate (86-100% at 14 dpi.  -The saliva of the infected individuals was not examined. |
|  |  |  | 20 |  |  | 1.68 x 10^7^ PFU/mL | ZIKVSPH2015 isolated from a human in Sumaré, Brazil |  |  |  |
|  |  |  | 20 |  |  | 3.55 x 10^6^ PFU/mL | Rio-U1 isolated from human urine in Rio de Janeiro |  |  |  |
|  | *Cx. quinquefasciatus* | Recife, Brazil | 20 | 5 to 7 | Artificial blood feeding | 2.3 x 10^6^ PFU/mL | ZIKVPE243 isolated in 2015 from a human in Recife, Brazil |  |  | -No infection. |
|  |  |  | 12-20 |  |  | 1.68 x 10^7^ PFU/mL | ZIKVSPH2015 isolated from a human in Sumaré, Brazil |  |  | -No infection. |
|  |  |  | 20 |  |  | 3.55 x 10^6^ PFU/mL | Rio-U1 isolated from human urine in Rio de Janeiro |  |  | -Low infection (5% at 7 dpi).  -No dissemination. |
|  |  | Campina Grande, Brazil | 20 |  |  | 2.3 x 10^6^ PFU/mL | ZIKVPE243 isolated in 2015 from a human in Recife, Brazil |  |  | -No infection. |
|  |  |  | 20 |  |  | 1.68 x 10^7^ PFU/mL | ZIKVSPH2015 isolated from a human in Sumaré, Brazil |  |  |  |
|  |  |  | 20 |  |  | 3.55 x 10^6^ PFU/mL | Rio-U1 isolated from human urine in Rio de Janeiro |  |  |  |
|  |  | Rio de Janeiro, Brazil | 30 |  |  | 2.3 x 10^6^ PFU/mL | ZIKVPE243 isolated in 2015 from a human in Recife, Brazil |  |  | -No infection. |
|  |  |  | 3-30 |  |  | 1.68 x 10^7^ PFU/mL | ZIKVSPH2015 isolated from a human in Sumaré, Brazil |  |  |  |
|  |  |  | 20 |  |  | 3.55 x 10^6^ PFU/mL | Rio-U1 isolated from human urine in Rio de Janeiro |  |  |  |
| Roundy et al. 2017 | *Ae. aegypti* | Salvador, Brazil | 9 | 3 | Artificial blood feeding | 4 x 10^4^, 4 x 10^5^ and 4 x 10^6^ FFU/mL | 41525 isolated from *Ae. africanus* in Senegal | Presence of ZIKV in saliva | Titration by focus forming assay | -Variation in competency for ZIKV among *Ae. aegypti* from the different localities.  -After artificial blood meals, strains FSS 13025 and MEX1-7 were refractory to transmission in mosquitoes from Brazil and USA  -Mosquitoes from Dominican Republic transmit the 3 ZIKV strains  -ZIKV strain from Senegal was the only strain capable of being transmitted by all mosquito strains  -Higher infectivity from murine blood meals than from artificial blood meals |
|  |  |  |  |  |  |  | FSS 13025 isolated from a human in Cambodia in 2010 |  |  |  |
|  |  |  |  |  |  |  | MEX 1-7 isolated from *Ae. aegypti* in Mexico |  |  |  |
|  |  |  |  |  | Blood feeding on ZIKV-infected mice | 10^4^-10^7^ FFU/mL | FSS 13025 isolated from a human in Cambodia in 2010 |  |  |  |
|  |  | Dominican Republic | 9 |  | Artificial blood feeding | 4 x 10^4^, 4 x 10^5^ and 4 x 10^6^ FFU/mL | 41525 isolated from *Ae. africanus* in Senegal |  |  |  |
|  |  |  |  |  |  |  | FSS 13025 isolated from a human in Cambodia in 2010 |  |  |  |
|  |  |  |  |  |  |  | MEX 1-7 isolated from *Ae. aegypti* in Mexico |  |  |  |
|  |  |  |  |  | Blood feeding on ZIKV-infected mice | 10^4^-10^7^ FFU/mL | FSS 13025 isolated from a human in Cambodia in 2010 |  |  |  |
|  |  | Rio Grande Valley, USA | 9 |  | Artificial blood feeding | 4 x 10^4^, 4 x 10^5^ and 4 x 10^6^ FFU/mL | 41525 isolated from *Ae. africanus* in Senegal |  |  |  |
|  |  |  |  |  |  |  | FSS 13025 isolated from a human in Cambodia in 2010 |  |  |  |
|  |  |  |  |  |  |  | MEX 1-7 isolated from *Ae. aegypti* in Mexico |  |  |  |
|  |  |  |  |  | Blood feeding on ZIKV-infected mice | 10^4^-10^7^ FFU | FSS 13025 isolated from a human in Cambodia in 2010 |  |  |  |
| Li et al. 2017 | *Ae. aegypti* | Haikou, China | 10-15 | 5 | Artificial blood feeding | 3 x 10^5^ PFU/mL | SZ01 isolated from blood of a patient who returned from Samoa to Chian in 2016 | Engorgement on infant mice and presence of the virus in their brains | Detection by qRT-PCR | -High infection rate  -High dissemination  -Transmission to infant mice confirmed |
|  |  | Ruili, China |  |  |  |  |  |  |  |  |
| Liu et al. 2017 | *Ae. aegypti* | Haikou, China | 18-30 | 5 to 7 | Artificial blood feeding | 5.45 log_10_ copies/µL | ZIKV isolated from a patient in China in 2016 | Presence of virus in salivary glands | Detection by qRT-PCR | -High infection rate  -High dissemination rate  -Moderate transmission efficiency |
|  | *Ae. albopictus* | Foshan, China |  |  |  |  |  |  |  | -High infection rate  -Moderate dissemination rate  -Low transmission efficiency |
|  | *Cx. quinquefasciatus* | Guangzhou, China |  |  |  |  |  |  |  | -Low infection rate  -No dissemination  -No transmission |
| Ciota et al. 2017 | *Ae. aegypti* | Poza Rica, Mexico | 12-30 | 4 to 7 | Artificial blood feeding | 4.6 - 8.9 log_10_ PFU/mL | ZIKV HND isolated from serum from a patient returning from Honduras | Presence of ZIKV in saliva | Titration by plaque assay and detection by qRT-PCR | - Freezing/thawing ZIKV impairs infectivity to mosquitoes at several doses of virus  High infection  - Transmission efficiency increases when viremia inoculated increases |
|  |  |  |  |  |  | 4.3 - 8.7 log_10_ PFU/mL | FSS 13025 isolated from a human in Cambodia in 2010 |  |  |  |
|  | *Ae. albopictus* | New York, USA |  |  |  | 4.1 - 8.9 log_10_ PFU/mL | ZIKV HND isolated from serum from a patient returning from Honduras |  |  | - Transmission efficiency increases when viremia inoculated is high |
|  |  |  |  |  |  | 4.2 - 8.6 log_10_ PFU/mL | FSS 13025 isolated from a human in Cambodia in 2010 |  |  |  |
| Pompon et al. 2017 | *Ae. aegypti* | Singapore | 20 | 3 to 5 | Artificial blood feeding | 10^5^ and 10^6^ PFU/mL | H/PF/2013 isolated from human serum in French Polynesia in 2013 | Presence of ZIKV in saliva and salivary glands | Titration by plaque assay and qRT-PCR | -Asian ZIKV strains may not be as efficiently transmitted as the American strains  -High rate of salivary glands infection in *Ae. aegypti* and *Ae. albopictus*  -*Cx. quinquefasciatus* is not a competent vector in Asia |
|  |  |  |  |  |  |  | BE H 815744 collected in the Paraiba State, Brazil |  |  |  |
|  | *Ae. albopictus* | Singapore |  |  |  |  | H/PF/2013 isolated from human serum in French Polynesia in 2013 |  |  |  |
|  |  |  |  |  |  |  | BE H 815744 collected in the Paraiba State, Brazil |  |  |  |
|  | *Cx. quinquefasciatus* | Singapore |  |  |  |  | H/PF/2013 isolated from human serum in French Polynesia in 2013 |  |  |  |
|  |  |  |  |  |  |  | BE H 815744 collected in the Paraiba State, Brazil |  |  |  |
| Kenney et al. 2017 | *Cx. quinquefasciatus* | Sebring, lab strain | 95 | 5 to 6 | Artificial blood feeding | 6 log_10_ PFU/mL | MR766 from Uganda | No saliva tested for transmission in the absence of dissemination | Titration and detection by plaque assay | -Very low infection rate at 14 DPI (1%)  -No dissemination |
|  |  |  | 36-43 |  |  | 4.0, 5.9 and 7.1 log_10_ PFU/mL | PRVABC59 isolated from human blood from Puerto Rico |  |  | -No infection  -No dissemination |
|  |  |  | 30-35 |  |  | 6.4 and 7.6 log_10_ PFU/mL | R103451 isolated from a patient who traveled to Honduras in 2015. |  |  | -No infection  -No dissemination |
|  |  |  | 23 |  | Intrathoracic inoculation | 6.7 log_10_ PFU/mL | MR766 from Uganda | Presence of ZIKV in saliva | Titration by plaque assay and qRT-PCR | -High infection rate (70%)  -No transmission |
|  |  |  | 33 |  |  | 6 log_10_ PFU/mL | PRVABC59 isolated from human blood from Puerto Rico |  |  | -Low infection rate (15%)  -No transmission |
|  | *Cx. pipens* | Chicago, lab strain | 20 |  | Artificial blood feeding | 6 log_10_ PFU/mL | MR766 from Uganda | No saliva tested for transmission in the absence of dissemination | Titration and detection by plaque assay | -Low infection at 14 DPI (5%)  -No dissemination |
|  |  |  | 38 |  |  | 6 log_10_ PFU/mL | PRVABC59 isolated from human blood from Puerto Rico |  |  | -Low infection at 14 DPI (10%)  -No dissemination |
|  |  |  | 28 |  | Intrathoracic inoculation | 6 log_10_ PFU/mL | MR766 from Uganda | Presence of ZIKV in saliva | Titration by plaque assay and qRT-PCR | -High infection rate (61%)  -No transmission |
|  | *Ae. aegypti* | Poza Rica, Mexico | 12 |  | Intrathoracic inoculation | 6 log_10_ PFU/mL | PRVABC59 isolated from human blood from Puerto Rico | Presence of ZIKV in saliva | Titration by plaque assay and qRT-PCR | -High infection rate (100%)  -High transmission efficiency (67%) |
| Tan et al. 2017 | *Ae. aegypti* | Singapore | 10 | 5 to 7 | Artificial blood feeding | 7.34 log_10_ TCID/mL | MR766 from Uganda | NA | Titration by TCID assay | - Infection and dissemination rates of ZIKV reduced among *Wolbachia*-infected *Ae. aegypti.* |
|  |  | *w*Mel-Sg | 14-15 |  |  |  |  |  |  |  |
| Duchemin et al. 2017 | *Ae. camptorhynchus* | Gippsland, Australia | 18-40 | 5 to 8 | Artificial blood feeding | 5.6 log_10_ TCID/mL | FSS 13025 isolated from a human in Cambodia in 2010 | Presence of ZIKV in saliva | Detection of infection and dissemination by qRT-PCR and determination of cytopathogenic effect of saliva followed by TCID assay for transmission assay | -Moderate infection rate  -Low dissemination rate  -Low transmission efficency |
|  | *Ae. notoscriptus* | Bellarine, Australia | 35-59 |  |  |  |  |  |  | -Moderate infection rate  -Low dissemination rate  -Low transmission efficiency |
|  | *Ae. aegypti* | Queensland, Australia | 38-48 |  |  |  |  |  |  | -High infection rate  -High dissemination rate  -High transmission efficiency |
|  | *Ae. albopictus* | Torres strait, Australia | 26 |  |  |  |  |  |  | -High infection rate  -High dissemination rate  -Moderate transmission efficiency |
|  | *Cx. annulirostris* | Lab strain, Australia | 32 |  |  |  |  |  |  | -No infection  -No dissemination  -No transmission |
|  | *Cx. quinquefasciatus* | Lab strain, Australia | 17-20 |  |  |  |  |  |  | -No infection  -No dissemination  -No transmission |
| Secundino et al. 2017 | *Ae. aegypti* | Lab strain PP-Campos, Brazil | 26 | 3 to 5 | Artificial blood feeding | 5 x 10^5^ PFU/mL | SPH/2015 from human serum in Brazil | Engorgement of a single mosquito on mouse ear | qRT-PCR and determination of cytopathogenic effect | -Successful transmission of ZIKV by infectious mosquito bite |
| Goertz et al. 2017 | *Ae. aegypti* | Rockfeller lab strain, Germany | 49-101 | 3 to 6 | Artificial blood feeding | 2 x 10^5^, 2 x 10^6^, 2 x 10^7^ TCID/mL | ZIKV Suriname strain 011V-01621 | Presence of virus in saliva | Titration by TCID assay | -High infection rate  -High dissemination rate  -High transmission efficiency |
|  |  |  | 96 |  |  | Co-infection of 2 x 10^7^ TCID/mL of ZIKV and CHIKV | ZIKV Suriname strain 011V-01621  Clone derived CHIKV 37997 |  |  | -High infection rate  -High dissemination rate  -High transmission efficiency |
|  |  |  | 49 |  | Intrathoracic inoculation | 2.8 x 10^3^ TCID/mL | ZIKV Suriname strain 011V-01621 |  |  | -High infection rate  -High dissemination rate  -High transmission efficiency |
|  |  |  | 48 |  |  | Co-infection of 2.8 x 10^3^ TCID/mL of ZIKV and CHIKV | ZIKV Suriname strain 011V-01621  Clone derived CHIKV 37997 |  |  | -High infection rate  -High dissemination rate  -High transmission efficiency |
| Gendernalik et al. 2017 | *Ae. vexans* | Colorado, USA | 148 | 5 to 7 | Artificial blood feeding | 7 x 10^6^, 1.3 x 10^7^, 1.7 x 10^7^ PFU/mL | PRVABC59 isolated from human blood from Puerto Rico | Presence of virus in saliva | Titration by plaque assay | -High infection rate (80% at 14DPI)  -Low dissemination rate (25 % at 14 DPI)  -Low transmission efficiency (7% at 14 DPI) |
| O’Donnell et al. 2017 | *Ae. vexans* | North Dakota, USA | 32 | 7 | Artificial blood feeding | 2 x 10^5^ PFU/mL | PRVABC59 isolated from human blood from Puerto Rico (fresh virus) | ND | Detection by qRT-PCR | -Low infection rate  -Low dissemination rate |
|  |  |  | 47 |  | Intrathoracic inoculation | 1.8 x 10^7^ PFU/mL | PRVABC59 isolated from human blood from Puerto Rico (twawed virus) | Presence of virus in saliva |  | -Moderate dissemination rate |
|  |  | Minnesota, USA | 28 |  | Artificial blood feeding | 9.2 x 10^6^ PFU/mL |  | ND |  | -Low infection rate  -Low dissemination rate |
|  | *Ae. aegypti* | Costa Rica strain, lab strain | 18 |  | Artificial blood feeding | 9.2 x 10^6^ PFU/mL |  | ND |  | -Moderate infection rate  -Low dissemination rate |
|  |  |  | 22 |  | Intrathoracic inoculation | 1.8 x 10^7^ PFU/mL |  | Presence of virus in saliva |  | -Low transmission rate |
